# Supplementary material for: A PPR Protein ACM1 Is Involved in Chloroplast Gene Expression and Early Plastid Development in Arabidopsis
Source: Int J Mol Sci. 2021 Mar 3;22(5):2512. doi: 10.3390/ijms22052512 (PMC7959153; doi:10.3390/ijms22052512)
Supplement: Supplementary file 1 [file ijms-22-02512-s001.zip › Table S1.docx]

**Table S1. A list of primers used in this study**

| **Primer name** | **Sequences (5**′**-3**′**)** | **Purpose** |
| --- | --- | --- |
| *ACM1*-RNAi-F1:  *ACM1*-RNAi-R1: | ATCCCCATGGCTCCGTCATTGGCACTC  CCCCCCATTTAAATGCGAACAGGCACTAAGAA | RNAi vector |
| *ACM1*-RNAi-F2:  *ACM1*-RNAi-R2: | AACCCTCTAGACTCCGTCATTGGCACTC  CCCGGATCCGCGAACAGGCACTAAGAA | RNAi vector |
| *ACM1*-rtPCR-F:  *ACM1*-rtPCR-R: | CTTCGGAGCAAAGATTA  AGCCATAGCGTCAGG | RT-PCR |
| *ACM1*-qRTPCR-F:  *ACM1*-qRTPCR-R: | TTTGCTGTATGCGTTTG  CACTGTTCGGTTTGCTT | quantitative RT-PCR |
| *ACM1*-GFP-F:  *ACM1*-GFP-R: | CGGGGTACCATGGCGGTTTCTGCAGG  CGCGGATCCATTCGGAGTCAACCCACC | Overexpression vector |
