# Supplementary material for: A PPR Protein ACM1 Is Involved in Chloroplast Gene Expression and Early Plastid Development in Arabidopsis
Source: Int J Mol Sci. 2021 Mar 3;22(5):2512. doi: 10.3390/ijms22052512 (PMC7959153; doi:10.3390/ijms22052512)
Supplement: Supplementary file 1 [file ijms-22-02512-s001.zip › TableS2 Splicing primer sequences.docx]

**File S2. The position of the primers used in splicing events**

Exons Primers exon and primer overlap

ndhA (ATCG01100)

TTAGAGTGAAAAGAGTTGGAAAGAAGTGGTTAATAATAGATTACCAAGGGAAATAGGTAAAAGAAATTTCCATCCAAGATTTAATAGTTGATCCATTCTTAGCCTAGGTAAAGTCCATCTTGTTGCGATAGAAACGAACAAAAACAAATAAGTTTTAGCTAATGTAATAAAGATACCAATTGTTGTTCCAAAAATTTGATCCCTTTGAAATAGCTCCAGAATAGATATATACGGAATAGAAATATTCCAACCGCCTAAGTATAGAACTGTTACAAATAATGAGGAAATTAATAGATTTAGATAAGAAGCAACGTAAAATAAACCAAATTTTATACCTGAATATTCAGTTTGATAACCTGCTATTAATTCTTCTTCCGCTTCTGGTAAATCAAACGGTAACCTCTCGCATTCTGCTAGGGAAGAAATTAGAAAAATGATAAAACCTATAGGTTGACGCCACAAATTCCATCCCCAAAAACCATATTTTGATTGTGCCTCAACTATATCAACTGTACTTAAACTGTTAGATAATCCTAGTCGGCGAGAACATTACTATTTTCACCGCTATTTCAGAACCGTACATGAGGTCTTGGCCTCATACGGCTCCTCGGGGGCCATAAATAAATCTAAGGACCAGATTATAATTTAGATGGATATGATGTGTTCTAAAATGGATTAAATATATCTGGGGTCCCGAATTATACCAATGGAATTCTGTCTGCTCAAATTCTAAGAATAAAAAACGCGCTTCGGAATTCATCTCACCCCTTTACAAATTTGAATTTCTATTTGTTGAGTAATAACTTAACCCTTTAATAAAAAACTCCTAAAAAAAAAAAGTATTTAAGCCCATCGTGGTTTTCGATTACGAAAAATAATTAGACATCCTATTTTTTTATTATTCATGACAGAAATTCGATTTTATTTTCGAAATATATGAAAAAAAAAAGAGCCTTGTTTCGTTCCTATTCTTCTTTCTTTTTTAGAAAAAAGTTGGTAGACTTATAAAAAATAAAGGATTATTTCGTTTCTGATAGTCATTACATTTGTCGGTGGATAGGAGCATACTCTGAATCGGAATCTTGGGGAGTACTGTCTGATCATTTCTACTAATTTAAAGCCCCAATTAACCTTCTTTTTTTTATCTTATGTTAGGCCTAAATATCCTTTTCAATTTGGTTAATCTCTATTACAAATTCTTTGTGTATTTTGGTGTTTCTAACCATCCACTCACTTTTACCTAATTGCCGCTCACTTTGTAATACATGTATGTTAATCTATATAACTGATAGTGAAAACGTCATCCGGTTACCGGTTAATATATTTAACCCGCTTCAAGTCAGGATGACTAATCAACCAACCTTGGGGTAAAGTGATTCTTACACTTATGTTTATTTCCGTTTAACCTTTGTACATAGGAAATGAGACTCATTTTTCGTTTTACTGCAAATTTCAGAGCAGTTTTTTTTTCACTCATATATAACTATCAAATTCCTTTTATTAATTATTTCGATTAATTCGAAAGATAAATATATATTCCGTTTTTTAACTGATTCGTCTAGAAGAAACGGAATAGTAAAAATAAACGTTTCTGTTTCAACGAATCGCACGTAGAGATATTGATAAAACACATAGAGTTAATGGTATTTCATAACTAATCGATTGGGCAGCAGCTCGCAGACCACCTAAAAAAGAATATTTATTATTTGATCCATATCCTGACATAAGAAGTCCAATAGGAGCAATACTTGAGATGGCAATCCATAAAAAAATACCGATATTGAGATCCGCTAAAACAAGGTGATTGCTAAAGGGAATTACTGAATAACTTAGTAAAATAGAGATAACTGCTATAGATGGTCCAATACTAAATAAAGGAGTATTTCCTCTAGATGGACGAAGATTTTCTTTGAAAAGTAGTTTTGTCCCATCGGCTAGAGCTTGAAGAATTCCCAACGGGCCGGCGTATTCAGGTCCAATACGTTGTTGTATCCCTGCAGATATTTCTCTTTCTAACCACACAATTACTAGTACACCTGTTATGATTCCCAATACAAGAGAAAATATAGGGACAAATATCCATATGAGTCCATAGACCTCTTTTAAAGATTCCAATTTAACAAAAGAATTTATAGTTTGGACTGCTGTTGCATAAATTATCAT

ndhB (ATCG00890)

CTAGAAGCTAAAAAGGGTATCCTGAGCAATCGCAATAATCGGGTTCATTGATATTCCTGGTATAGTAGATGCTATCACACATACAATCATACTCAATTCGATGGAATTGTTTGATCTTAAAGGGGATATTCTATAATTTCGCATGTGAGGGGTTATTTCTTGGTTTCGTCCAGTCATTAATAACTTGATTATTTTTAGATAATAGTAGATAGAAAGAACGCTCGTAAGGAGTCCTATTGAAACCAAGAAATATAGGCCTGCCTGCCATCCACACCAGAATAAATGGAGTTTTCCAAAAAAACCTGCTAGTGGAGGAAGACCTCCTAGGGATAAGAGACATAGAGCTAAAGAGAGAGCCAAAAAAGGATCTTTTGTGTATAATCCTGCATAATCTCGAATGTTATCAGTTCCGGTACGTAGACCAAATAATATAATGCAAGCAAAAGTTCCTAGATTCATGGCGATATAGAACAGCATATAAGTTATCATGCTCGCATATCCACCATTTGAGTCTCCAACAATTATTCCAATAATTACATATCCGATTTGACCTATGGACGAATATGCAAGCATACGTTTCATGCTTGTTTGAGTAATAGCAATGAGATTCCCGAATATCATGCTAAGAATAGCTAGGATTTCCAGAAGAAGATGCCATTCATTTGATGAGAAATAAAAAGGAATATCGAAAATTCGAGTGGCTGAAGCTGAAGCAGCTACTTTCGAAGTAACAGAAAGAAAAGCAACGACTGGAGTGGGAGAGTCAGAGTCGAAAAGAGGATTCCTCACTTCTTTCTCTCATTCAAAACCGTGCATGAGACTTTCATCTCGCACGGCTCCTAAGTGATAAAAGTAAAGAAGAACTCATCTTCTTTCTTTTTTGATTACTTTCCTCGCGTATGTATAAGATCGAATCCTTTCTATTTCTAAAACGGATTACTAATCCTTAACTTTTCGAGGAATCCTTCATCAGTGGTTGTGAATGACTGATTTTTCTCAATCTTTTCGACCTTGGTTCCGTAGGAGCACGTCCGAAAGATTGAGAAATGGAACCATCTGATTTGATTCGTTCTCAATAGCCATGAGATTATCATCTTAGGGTGATCCTTTTGTCGACGGATGCTCCTATTACACTCGTAGTCTCTGAAGGATGAGAACCAACTATGTAGCATCTACATCGAGAATTCAAGTCTTTCTTGTATACGTCATTAGTCCGATCCTTTGCAGGAACTACCCGTAATAACAAACTTGCAAAATGGATCCGTTTATCATAAAGAGATTCGTTGTTCCTGACCCTGCTTCACCTTAATTGTTATTTGAACAAGTCAAAGTTCTGTCTTGGTCTGCGTGGGGATAGCATTTCTCTTCTGCATGTCCATAGAGTTTTGAAAAATCCAAACATCTCAGAGATAGATAGAGAGGTAGGAATTTCTCAAACGAACCGCACTCCTTCGTATACGTCAGGAGTCCATTGATGAGAAGGGGCTAGGGAAAGCTTGAACCCAATTCCTACAGTGATGAATATAAGCGCAATTGAAATTCCTGGGGAGTTATACATTTGTGTATTGATAAGACCATTCACTATTTCTTGAAGCTCAATCTCTCCCCCGGATGAACCATATAGCCAAGAGAAACCATGAACCAGAATAGAAGAGCTTGCCCCACCCATGAGTAAATATTTCATAGTAGCCTCATTAGATCGTATATCTTTCTTGGTATATCCAGATAATAGGTAGGAGCATAAACTGAAACATTCTGGAGCTACAAAGATAGTTATTAAATCGTTAGCACCACATAAAAACATTCCTCCTAGAGTAGCTGTTAATATGAATAACAGAAACTCTGTTATAGCCAT

ycf3-int-1 (ATCG00360)

TTATTCGAAGCGCCTCGTGATCGTCAACCAATTCTGTGCTTCAATATAATTACCAGGAGTAAGCGTTATAGCCTGTTTCCAATACTCAGCGGCTTGAGCGAACCAAGCCTCCGCCATTTCAGAATCTCCTTGTTGAATGGCCTGTTCTCCACGGTCGGAATAGGCGGATCAATTCCCTCCCTGAGAACCGTACTTGAGAGTTTCCTACCTCATACGGCTCGACAACCAACTCTTTTGTTTTGGTGTACCAGTTTTTTAACTTTAACCTACTTTGAACTTTATATCTAATTGAATGTCTAATTGAATGAGATTTCTTATAGATTTTTTGTTTTTCTTGGATTAAACAAAAGAGAGTAATTACATGAGTTTCAAACTTTCATTTTGATTTAATTAATATATTAATTAATATAATAAGTTTTATCTTTTCTCCTACCTTCAGAAAAAAAAGCATGTCCACTGTTATTAGATATTAGAATTTTCTGAAAGGTAACTATCCCGCTTTCATATAAAAATTTATATAGAATCTTTGAAAAAGACTTTTTCATACTTCATAAAAAAGAAAAAGACTTACTGTCTTTAGGATCTGATGCTACACCGCTGCTCAATACCTTCGGGGATTACTCTATTACATAAGTAGATTCCTAAGATTTATCTCATATTATGATATAAATAAACAGCTCTTGTTGTGTCGGTCCAAAACCTTTCCAATTGATCTTTACGGTGCTTCCTCTATCAATTAAATCTTTTTTTATCCATAGAAAAGAAAGTATTTAGGCATATCTAGTCTTCACTTCATATTTCGATGGATGAAGTTTAGTTATTTGCTACAGCTGATAAAAATCGTTTTAGGCGATGCTTATGTAGAAAGCCCTTTTTTTGTGTTTCTAGTATTTTATTGACTAGCGTTCGTTCTTTTTTTCTATAGTGGAGATAGTCGCACGTAATGACAGATCACAGCCATATTATTAAAAGCTTGTGGTAAAAAGGGGTTTCGTTCTAATGCCCGAAAATAATATTCTAAAGCTTTGGTATGTTCCCCATTACTTGTATGGATAAGGCCTATATTATAGAGTATATAACTTCGATCATAGGGGTCAATTTCTAGTCGCATAGCTTCATAATAATTCTGTAATGCTTCCGCATAATTTCCTTCAGATTGAGCCGACAT

clpP (ATCG00670)

ATGCCTATTGGCGTTCCAAAAGTACCTTTTCGAAGTCCTGGAGAAGGAGATACATCTTGGGTTGACATATAGTGCGACTTGTCAGATATATCGGGCTATATGGGATTTCCCCATTTTCTCCCTCGATCGAGATATCCTCTGTTTCGCTCAAAAAGATTGATTGAATTATCAAAAATTTGTAACGCGAAGCGCAAAAAATAAAGTGGAATTAAATGCAGGGAGTATTTAGATTGATATTAGATTATCAAAATTTTTTTATGGTTTACGTGATCGGACTAATAAAATGAAGTATCCAGGCTCCGTTTAGCAAAAACCCAATTGATAATTAATATATAATATTTTTATAATTACTACTTCTATTATATGCAAAATTATGATAAAAACTCTATAAAAAAAATTGAAACAGGGCGATCTAAAACTGTTGCTCAAATCAAATACAAATAATATATAATATAATTCAAAATTTGTTGACTATTTGACAGAAGACAAGTGAAAGAACTGAATTGAAAAAAAAGAAGGAGTAGTAAAAAAAGACGCGGTATTTGGTTCATTTGTCCTATATGTGCAAATCAAAATCGGGCAAATTTTTCCTTTTACTCGGGGTAGAGCATAAACCTAAAAAATGGAATAAAAAAAGGAAGAAGCCCGTTCAGGAACAAGAAAAAACCATCGCCATTTCAACTGAATCTCGATGAAAAAAAAAAATATCAATGAATCAATCATTTTTTTAGAGGATTTTAATATCTAATAAAAGGCGCCAAAACTCAAAATTTCTTTTACTTTTGGGGCAAGCCCTTCCGTTATAAGTTAGAAAGAAAAACCCTCTATGAAAAAAGGGTAGGTTGGAATCAACACATTGATTTTTTCACAATTTTTATTGAACCGTATGCACCAAAAGGTGCCTGTACGGCTCCTAAGGAATAAAAAATTATCCTAATCAACCGACTTTATCGAGAAAGATTATTTTTTTTAGGCCAAGAGGTTGATACCGAAATCTCGAATCAACTTATTAGTCTTATGATATATCTCAGTATAGAAAAGGATACCAAAGATCTTTATTTGTTTATAAACTCTCCTGGTGGATGGGTAATATCTGGAATGGCTATTTATGATACTATGCAATTTGTGCGACCCGATGTACAGACAATATGCATGGGATTGGCCGCTTCAATAGCATCCTTTATCCTAGTCGGAGGAGCAATTACCAAACGTATAGCATTCCCTCACGCTTGGCGCCAATGAGTTTTTTTATTTTCGAGAAAAAAATACTATGCCTTCGCCATTTGAAATATGAATTAGTTAAGTAATAATAGCATGGCACTTCGAATTCAATATGAAATTTTTTAGATTAAAAAAAAATTCGATTATATATTGAAAGAGTAGTATGAGATAAGGAAGAGGTTTTTCAAATGATATCTTACCTATTCGGGCACATTTCAGCGTCACAAACTTTGTTTTCACACCGTAAAAAAAAAAAAAGACACTTTGGGATTGCTGAATCATCGACGAATCAAAACAATGATATATAAAGCAACGGAACCATCATAGTATTTTTTTAACTCCTACAAAAAAAAGAAGGATGGTAATTGGATGATTTAAGGAAAGGTCAAAAAAGTCAATTCCATTGTGGAGCCGTATGCAATGCACAAAAAAAGCCTGTACGGTTATTCAAATTTATCTATTTTTTTTTTTTTGTTATCCCGTCTCATTCTGCGAAATAGAAAAACCTTTTCTATTATATCATCAGGGTAATGATCCATCAACCCGCTAGTTCGTTTTATGAGGCACAAACGGGAGAATTTATCTTGGAAGCGGAAGAATTACTTAAACTTCGCGAAACCATCACAAGGGTTTATGTACAAAGAACGGGCAAACCTATATGGGTTATATCCGAAGACATGGAACGGGATGTTTTTATGTCAGCAACAGAAGCCCAAGCTCATGGAATTGTTGATCTTGTAGCGGTTCAATAA

trnG (ATCG00100)

GCGGGTATAGTTTAGTGGTAAAAGTGTGATTCGTTCCTTTAATCCCTTTAATAGTTAAAGGGTCTCTCGGTTTGATTAATCTTCCGATCAAAAATTTTATTTCTTAAAAGGATTTAGTCCTTTCCCTTTCAATGAAAGATTCAAGGAAGATTATAGATTCTCGTAATTTATATCCAAAGACTCTAATCAATTGTAAATTTGGATTATGAAATTCCGAAACATAATTTTTGAATTGGATGACTATTTACAATTCAATAAGTATAACAAGAGGATCCATGGATAAAGCTAGAAAAGTGTCTTTCTAATCGTAACTAAATCTTCAGTTCTATTCATTGTTTGGTATAGAAAAATTGAAGCAAAATAGCTATTAAACGAGAACTTTGGTTTACTAAAGACATCGACATATTATATTGTTTTAGCTCGGTAGAAACCAAATACTTTTCCTAAGGATTCCGTTAAATAGAAATAAAGAACGAAGTAACTAGAAAGATTGTTCGAGTTGGCCCGATCTATCTTCTAGAAGGATCATCTAGAAAGCAAAATTTTCTGTGAAAGTACCCAGACGGAAAAAAGCTCACATAGATGTTATGGGTCGAATTTTTATTTCGTTCCCGTCTTTTTTTATTTGGGAATTTCTCCATCCATCATAAAGGAGCCGAATGAAACCAAAGTTTCATGTTCGGTTTTGAATTAGAGACGTTAAAAATATAAAAATGATCAATCGACGTCGACTAAAACCCTTAGCCTTCCAAGCTAACGATGCGGGTTCGATTCCCGCTACCCGCT

rpl16 (ATCG00790)

CTATTCAGAAATAATGAATTGGGTTTTTATAGGCATTTTTGATGCCGCTATTGAAATAGCTTTTCTTGCTATATTTTCGGGTACACCACCCATTTCATAAAGGATTTTACCTGGTTTAACCACAGCTACCCAATACTCAGGGGATCCTTTCCCAGAACCCATACGCGTTTCCGCAGGTCTTACTGTAACTGGTTTGTCTGGAAATATACGTACCCAAATTTTTCCACCACGTCGTACATTTCGTGTCATTGCGCGTCGCCCTGCTTCTATTTGTCTAGATGTGATCCAAGCGGGTTCAAGTGTTTGAAGAGCATATCTGCCAAAACAAATACGATTCCCACGAGAAGATATTCCTTTTAGTCTTCCTCGATGTTGTTTACGAAATCTGGTTCTTTTTGGGTTATAGTTGATGGTTCTAAATTAGAAATTCCATCTCTACTACAGAACTGAACGTGAGAGTTTCTTCTCATCCAGCTCCTCGCGAATAAAAGGACTAAAAAAGCTTGTATTAAGATATAGTTAATGATTAATTCTATTAATCATGATATTCAATTTCATCTGATCTCTTCTAAATTTGTGTATGTCTTTTTCGAAATGGAATCCAAGATTAATTCTATTTATTTAAAAATAACGTAACGTAATATCATCATCTCGAATGTCGTTTTTGTTAGAATATTCTAACAAATCCTTATTTTCTTTTATCTTTTTAATTTTTGTTTTTTATTACTTTTATTAAAAAAAAAGTATTCGCCGGCGAATATTTACTCTTTCAATATCTATATTAAGTTTGTTGTTTATCCCAGGAGATCTCTGAATAAAAATAAGAATAGATAATAATCTGGTTTATTCCGCCATCCTGTCCAATGAATTACTAAGATTTATTTTTCAATAGAATCCTCTATATTCATGGGTTCCGTCGTTCCCATCGCTTCTTGATTAATCATTAGGCCCGAATTCTACAATGGAGCTCTTATATGAAATCGGGAATTTCATTTTTTTTATTTGTTTTTGAGTCAATTTTCTTAGTTTTTATTGGCTCAAGGCTCTTAATTTTTTTTGTTTTCGGAACAGATTTATCTAATTATTCTGAATGAATCTGTATTGATGCTTTATTACATTGCTTTTCTTACAGTGACCTCATAGACTTTCCAAATTGGAATAATATATCATTAATATTCAATTTTTTCTCTCTTTCTTTCATCCTTCCACTGAATCCGCATACTTTTCGATTACCTTTCATAACTTATAATTATAGTTCTTTATTCTTTTTTTTTTTCAGTTGCTACAATAATATGATCAATCCATCATATCTTGACTGATTTTTTTATCCAGATAATGTGAAGCAATGAGTTGCTTAGGTTATTTATTAATGCTATAGTTATTAGTTTCTGTTATAGGTAAGTTCTTTTTTCTTTTTTTTTTATCTTTATCTAATCCTAAACCAACGAGTCACACACTAAGCATTTTTATAGGCATTTTTGATGCCGCTATTGAAATAGCTTTTCTTGCTATATTTTCGGGTACACCACCCATTTCATAAAGGATTTTACCTGGTTTAACCACAGCTACCCAATACTCAGGGGATCCTTTCCCAGAACCCATACGCGTTTCCGCAGGTCTTACTGTAACTGGTTTGTCTGGAAATATACGTACCCAAATTTTTCCACCACGTCGTACATTTCGTGTCATTGCGCGTCGCCCTGCTTCTATTTGTCTAGATGTGATCCAAGCGGGTTCAAGTGTTTGAAGAGCATATCTGCCAAAACAAATACGATTCCCACGAGAAGATATTCCTTTTAGTCTTCCTCGATGTTGTTTACGAAATCTGGTTCTTTTTGGGTTATAGTTGATGGTTCTAAATTAGAAATTCCATCTCTACTACAGAACTGAACGTGAGAGTTTCTTCTCATCCAGCTCCTCGCGAATAAAAGGACTAAAAAAGCTTGTATTAAGATATAGTTAATGATTAATTCTATTAATCATGATATTCAATTTCATCTGATCTCTTCTAAATTTGTGTATGTCTTTTTCGAAATGGAATCCAAGATTAATTCTATTTATTTAAAAATAACGTAACGTAATATCATCATCTCGAATGTCGTTTTTGTTAGAATATTCTAACAAATCCTTATTTTCTTTTATCTTTTTAATTTTTGTTTTTTATTACTTTTATTAAAAAAAAAGTATTCGCCGGCGAATATTTACTCTTTCAATATCTATATTAAGTTTGTTGTTTATCCCAGGAGATCTCTGAATAAAAATAAGAATAGATAATAATCTGGTTTATTCCGCCATCCTGTCCAATGAATTACTAAGATTTATTTTTCAATAGAATCCTCTATATTCATGGGTTCCGTCGTTCCCATCGCTTCTTGATTAATCATTAGGCCCGAATTCTACAATGGAGCTCTTATATGAAATCGGGAATTTCATTTTTTTTATTTGTTTTTGAGTCAATTTTCTTAGTTTTTATTGGCTCAAGGCTCTTAATTTTTTTTGTTTTCGGAACAGATTTATCTAATTATTCTGAATGAATCTGTATTGATGCTTTATTACATTGCTTTTCTTACAGTGACCTCATAGACTTTCCAAATTGGAATAATATATCATTAATATTCAATTTTTTCTCTCTTTCTTTCATCCTTCCACTGAATCCGCATACTTTTCGATTACCTTTCATAACTTATAATTATAGTTCTTTATTCTTTTTTTTTTTCAGTTGCTACAATAATATGATCAATCCATCATATCTTGACTGATTTTTTTATCCAGATAATGTGAAGCAATGAGTTGCTTAGGTTATTTATTAATGCTATAGTTATTAGTTTCTGTTATAGGTAAGTTCTTTTTTCTTTTTTTTTTATCTTTATCTAATCCTAAACCAACGAGTCACACACTAAGCAT (AGCAATTATATCAAAGGA other gene sequence)

petD (ATCG00730)

TTAAAAAAGACCTAAAGTTAGAGATTTATCAATCGGTAATGTTGCTCCAATACCTAACCACAGGGCCGCCGCGGTGCCAATCAAAAAGACGGTTGTCGCTACTGGACGACGAAATGGATTTTGAAACTTATTAACATTTTCCAAAAAGGGTACGGTTAATAATCCCGCTGGTACTGAAACCATTAAAAGAACACCCAATAATTTGTTAGGCACTGTACGAAGTATTTGAAATACAGGAAAGAAATACCATTCAGGTAATATTTCCAAAGGAGTTGCAAAAGGATCCGCAGGTTCACCAATCATTGATGGTTCTAAAACCGCTAAGCCTACGTTACAGGCAATGGTACCAAGAATAACTACTGGAAAAATATATAAAAGGTCGTTGGGCCATGCGGGTTCCCCGTAATAATTGTGACCCATACCTTTAGCTAATTTAGCTCGTAATACAGGATCATTCAAATCTGGTTTTTTTGTTATTGGGATAGGTGAATTCTTATGGATCCATCCCCCGAGGGAACCGGACATGATAATTTTTTATCATCCGGCTCGAGCAAGAATTAACCAAACTAAATGAATACATACAAAAAATATAATTTAATATGTAAAAATTAATTATCCGATTCCAAAGTTATTGCAACTAAAGATTCAAGAAATTCCATTCTTACAGGTAAATGCTCAACACCCACGTAAGCTTAAAAAGTTTATCATGATTAAGTGCTTTCTGGGTCGTCTCATACCTTATAAATTCGTCCTTAATCTCAACAATTATATCGAGATTTTTCAAATACAAAGGATTTACTTGTTACTAATGGAGTCTACCCTCTACTCTTCATTAGATCCTGTATTCACTCCGATAGTATCATAAATCGAGTCGATGCAGAGAAAATGAATGCATTTACATACTATTACTATTAATAATTATTTTCTTTTTTTTTTTTAGTCCAAAATAACTAAAAACATAATCAGGATTTTGTTCCATCCCTTAATCTACTGGATTTTACGGAGCCCACTCATCCACGACAGCGTCGGGTACGATCATAGAAAAGATTCTCTTCAAGTGAACCAGCCTATCCCCTGTATGGGGCTTACACAGTGGTTGGAACAAAGACACATTTGGTTGTGAATTCCAATGTAGCAGATAAAGTAATATTTCTGCACGGCCCGATCAATAGTTCAAGTCACACACTCCCAT (CGAATTATTTGG other gene sequence)

rpoC1 (ATCG00180)

TTAGGTATCATATGAACAGGCTTGAGAAAAACCTTGTATAGCTTCCTCGATTTCTCGATAAAAAGAAATATGACCAACTGTGGTTCGAATATATATACAAAAATTTTCTTTTTTTACACTTCTTACTATTAGATAGTGTGCATAAATCTCATGATAGTTACCAAAAGATTCATAGTGAACTTCGATAGGAACTTCTCTTGAAGCAATAACGCGTTGATCTAATTGCCACCGAAGCCACAAAGGACTATCTAAATTGATTTTTTTCTGTCGATAAGCTCCAATTGCATCATAGGAATTGCAAAAAAAGGGTTCTTTCGTATACTTATAGTTTGTTTCGTAAATTCTTTCATTTTGATAGTTTTTTCGATTACATGGATTATATCTGTTTGCACAAATACCTCGACGAGTGCCGCTCGTTAATACATAGAGTCCAATAAGCATATCTTGAGTCGGTACCGAAATGGGATCTCCAATAGCTGGAGATAAGAGATTCATATGAGAAAACATAAGTAAACGAGCCTCTGCTTGAGCCTCTAAAGATAAAGGCACATGAACAGCCATTTGATCCCCATCAAAGTCTGCATTGAACCCCTTACAAACTAATGGATGTAAACAAATAGTGCGTCCTTCCACTAAAATAGGTTGGAATGACTGTATGCCTAATCTATGTAGGGTAGGTGCTCTATTCAGTAATACGGGATGCCCCTGCATGACTTCTTGAAGGATTTCCCAGACAATCGGCTTTTTTTCACGAATTTGACTCTTAGCAACTCCTATGTTCGAAGCCAGGTGTTGTCTAATTAAACCACGAATTACAAATGTCTGGAAGAGCTCTATTGCTATTTCGCGAGGCAATCCACAGCGATGTAATGAAAGTGAAGGTCCAACGACAATCACCGAGCGCCCCGAATAATCGACCCGTTTCCCAAGCAGAGTCTCGCGAAATCTTCCCTCTTTGCCTTCAATTACATCTGAAAATGACTTGTAAACCTTATTATGACCATCCCTCATGGGTTGTCCACGGATTCCATTATCAAGAAGTGTATCCACGGCTTCTTGTACCAATTTTTCCTGACACATTACTAATTCCCCCGGTGTAGATCTACTTGTTGTTAATAGATCGGTAAGAGTATTGTTCCGATAGATAACTCTTCTATAGAGTTCATTAATATCTGAACTCATTAGTTTACCCCCTTCTATCTGAATGATGGGTCTCAACTCGGGAGGAAGAACCGGTAAGAGACATAAAACCATCCATTCGGGTTCTATATTTGTTCGAATAAAATGCTTAGCTAATTCCATACGTCTAACTAAAAAATCTTTTCTTCTTACAATTTTTCGATCTTCCCATTCATTCCCCGTAGGCCCTTCTTCTCCTAATTGTTTCCATTCTACCAACGAATTTTCTATAATAATTCGCAAATCTAAATCGGCTAATTGTTCTCGGATAGCACCCGCCCCAGTAGAAATTTCTCGATTTCGAAATATATCGAAACCTTGAGTAGTAAAAAAAAGTGGGATGCTGTATTTCCAGGATTGAATTTCATATTCAAATGAACCTCGTAATCGTAAGAAAGTAGGTTTTTTAGTTATGGGCCTAGCAAAAGAAAAATTGGGATAGGGTCCACTATAAGATCTCCCCCCTCAAAACCGGACATGAAAGTTTCCTCTCATCCGGCTCAAGTAGTTATACCAAATAAAGAAAGGGGTCTCGCTTTCAAATTTCATTTTATAAAATAAAGTGAAAAACCCAAAAAGAATCTACGCCTTACTCAAGTTCTCAGTGCAAACCAACCAACATTTCATTGATTCAATTAATTCTTCTTTGATTTCTATTTAAATTCTTTAGTGAATTAAAAATTACGACAGAAAAAAATGTCAAATTCTTGAGTAGTCTACTTCCCTTCGAATGCCGGAATACTCTTTACCTTAAGTGAAAGGAATGCCTTAAAATTCATACGGGATTTATTTGTCTATGTATTGTTCCATTCGATCTTTTAGGTCCTGCGTCACCTCGATGGTTATGCCACAATATTCTTAAAGCTTATATGCGATGTATAGACTTCTGCAACCATGACATATTTGTTTACTTTATATAAAAATAAAATAATATAAAACTATAAAAAACCAAATTTATTTTCTTTTAGAAAGATAAGGGAATGCGTAATTCGACAAAACAAGGTCTTCTTTTCACGAGGTACGCCTATCAATTTGAATTTCTGAATCGACCATAGACCAATCGCCTTTTTTTTTATTTGGGAGTATTGAATACACCCACAATTCTGAGCTTCATGTTACTCTTTTCAAGAGACATGTCAGATCGAGGGCATCCCAAATTGATTGAAGGGGATGAGAGTTTATCATTCTTAATCTGTAAAAGAAAAATTTCGATCAAATCACACATCGCAGTATACTAGGCCTTCTAATTCTTTAAGAGGTTTATCTAAAAGATTCGCAATATAACTAGGAAGACGTTTCAAATACCATACATGAGTTACAGGACATGTCAGTTTTATGTATCCCATTTGATATCTTCGTATCCGAGAATCAACAAATTCAACTCCACATTGTTCACAAAACTTTGGGTCTTCTTTTTCATCTCCGATCACTCGATAATTTCCACAAGCGCAAATTCCACTCTTTATAGGTCCAAAAATCCTTTCACAAAATAATCCATCTTTTTCCGGTTTATTGGTTTTGTAATGAAAAGTATAGGGTTTTGTCACCTCTCCAACTATCTCTCCATTAGGTATTATTTTAGTGGCCCAAGCACTTATTTGCTGAGGAGAAACTAAGCCAATTCGGAGTTGTTGATGTTTATACCGATCGATCAT

pet B (ATCG00720)

TTATAAGGGACCAGAAATACCTTGCTTACGTATCATTAGGAAATGCATTAACATAAAGACGGCCGTAAGAAGAGGTAATACAAAAGTGTGTAAACTATAAAAACGAGTCAAAGTGGATTGTCCAACACTAGCACTTCCGCGTAATAATTCTACAAGAGGTGATCCTATTACCGGAATAGCGTCAGGTACACCTGTTACAATTTTGACCGCCCAATAACCAATTTGATCCCAAGGTAAAGAATAACCTGTTACACCAAAAGATGCGGTCAATACACCCAGAACCACACCAGTAACCCAAGTTAATTCGCGAGGTTTTTTAAAACCACCGGTGAGGTATACACGAAATACGTGCAGGATCATCATTAGGACCATCATACTTGCCGACCATCGATGAACTGATCGGATTAACCAACCAAAGTTAGCTTCAGTCATTATATATTGAACAGAAGCAAAAGCTTCAGTAACGGTTGGACGGTAATAAAAAGTCATAGCAAATCCCGTAGCTACTTGTACTAAAAAACAAGTAAGGGTAATTCCGCCTAGACAATAAAATATGTTGACATGCGGAGGAACATATTTACTAGTTATATCATCTGCAATCGCCTGAATCTCAAGACGTTCTTCGAACCAATCATAAACTTTATTGAGATAGGTAAACCAAGGTTACTCCCCCTCCAAGAACTGTATATGAGAATTTCATCTCGTACAGCTCAAACAAAAAAAGACCCAAATACTGATTGAAACAGATATGGAATATAAAGTTTTAAACTTGTCTCTCATTCAATATTATTTTTTATTATTTAAAGATATGAAAGAGTCAAAATGCGAAAGCTCGTCTTTGTGGTTAAATGCCAAAAAATCAAGTAAGCTCATTCGTCTTTATGTTGTGTTGATCGTTACAGGCCTCTTGAATCTTCTAGATTACCTATCTTTATTGTCTTTTTTTATTTGGTATTTGTATGGAACGGGGACAGGGATTTCTTCTTGTTTTCTTTATTATTGATAGGAATTCTCTTGTTAAGACCCTACTTAACTAATCAATTGAAACCTCAGATTCATACGAGAACCACGATAATTTAATGAAACCTTCAAAGTCCAAAGTTCACTGAGTGAGAACCATTGGATCATCACTTATTCAATAAAAAAAAAGCTATAATGACTAAAAACATAAAATACAAAGAAGCGTTTGTCCTTTGATCCAAATAAGAGTTTAAAAGCAGAAAATTTTTTTGATTTATTAAAGTTTATAAGATAGAACGGAAAGAAGTCCGGCTACGAGGTTTGAGTATTTAAGTATGAATCATAGTTCGAATACTTTGTGATCTATTTGATCTATTTCGTGCTCCAGATACTCGAATGAATATCCGACGAAGTAAAACCATCTTGATAAAGATGACAGACCCCATTCTCTGTACTATCCATAGGGTCAATTCTAACAAGTCACACACTCAT (GTATATTTCCGGAAT other gene sequence)

trnL (ATCG00400)

GGGGATATGGCGGAATTGGTAGACGCTACGGACTTAATTGGATTGAGCCTTGGTATGGAAACCTACTAAGTGATAACTTTCAAATTCAGAGAAACCCTGGAATTAACAATGGGCAATCCTGAGCCAAATCCTGGTTTACGCGAACAAACCGGAGTTTACAAAGCGCGAAAAAAGGGATAGGTGCAGAGACTCAATGGAAGCTGTTCTAACAAATGGAGTTCACTACCTTGTGTTCATAAAGGAATCCTTCGATCGAAACTTCAAATCAAAAAGGATGAAGGATAAAAACCTATATTGTATAAATTTAGGTAACACAAAACGATCTCAAAAATGACGACCTGAATCTCGATTTCTATTTTTTTATAAACAAAATCAAAATGTTGTGAATCAATTCGAAGTTTAAGAAATAATATTTCTTGATTAAAATAAAATTATTCACTTCATAGTCTGATAGATCCTTGATGGAACTTAATTAATCGGACGAGAATAAAGATAGAGTCCCATTTTACATGTCAATACTGACAACAATGAAATTTATAGTAAGATGAAAATCCGTTGACTTTTAAAATCGTGAGGGTTCAAGTCCCTCTATCCCCA

rps16 (ATCG00050)

ATGGTAAAACTTCGTTTAAAACGATGTGGTAGAAAGCAACGTACGACTTGAATTGAAGGACATTATCTGCTGTGGATTTTTTCATCCGCCACTTTTATTTTTATATTAGGAATATAGGTGCTCTTGGCTCGACATTTTGTATTCTATTTTACAAGAGTCCTACACTTTTTTGTAATATAAAAAAGAGTACAGGATGGAGCTCGAGGAGAATAGAAAGTTTTTAGTCCTTTCGCAGGAGTAAGGATTTAGGGTTAGTGCGAATTAATAAGTTGGAACAACTTCGTAAGTCTTTTTATATTGAAAAAAGTCCTTTCAAGCAATTTTACAATGGAAAAGGAAATTTTCTTAAAATTGTAAAATTCTTTGAATCAAAAGTCTATCATGCGTGAATCAAGCGTTTGTATGATTCTTTGTATAGAAAGAACAGAAATCATAAACAAAATAAGGGGCTTGTTGCTGCCCTTTTTTAATAAAACGATTCAATATCACCGAAGTCATGTCTAAACCTAAAGATTCAAAGTAAGGATAAAGAATCTTGAAAAAAGGAAATCCTGTTTTCAATTGTTTGAAAAACTAGATCAAACTGAAGAATCAAAATTGATTCTAAAATTTGCAATGAGACAAACAAAAAAAGGGCTAGAGACTACTCAATAAAAAAAGTACTTAAGGATTCTCCGGTGAGATATTTGAGAGTTGTTTAACTTGAGTTACGAGAGTACGAATGTTATGAATGCTTTTTATGGAAAAAATATTTAGGGTTTTAATACTGGCTAATTGATTTAATGTTTTTATTTCTCTATTTAATATTTGAATTTACTATTTGAATTTTTTTTCTCGAGCCGTACGAGGCCAAAACCTCTTATACGTTTCTAGGGGGGGTATTATTCATATACATCTATCCCAACGAGCCGTTTATCGAATCCTTGCAATTGATGTTCGATACCGAAGAGAAGGAAGAGATCTTAGTAAGGTGGGTTTTTATGATCCCATAACTAATCAAACTTTTTTAAACCTTTCTGCTATTCTCGATTTTCTTAAAAAAGGAGCTCAACCAACAAGAACAGCTCATGATATTTCAAAGAAGGCTGGGATTTTTACGGAATGA

trnV (ATCG00450)

AGGGCTATAGCTCAGTTAGGTAGAGCACCTCGTTTACACGTGCGCCAAAGTTTTTCAGAGGAGTCCATCACGCAATCAAACTAATTGATTGATCTTATTAAGAAATCGATGTCTTACTCCATGACTTTTTTTTAGGAAAAAAGAGGAGAACAACAGCCTGACATTAGGTCCTATTAAAGTACCCCGTTAGGTAGGGAATGAATAGAACCCATCATTGATTTGAGATATTGATAGGGTGAATACCCAGTCTACTCAATGCTAGGTAGAATGAGTATAAGGAACTCAAAAATGATCTTTTCGTCCTATGAACCTTAAGGTGTATCAAGTTTCATGTTTGATTTTTTAATCAGGATGTTAGAGACTATATTTAACTTAAGTTGATCTAGACCAAAAGCAAACCTACGTCAAGAGAACCCTTCTTTGAAACACTTTGGTAGTTCTTCTGTATTGTATTAGAATTCAAAAATAATCAATCAGAGTACTTGGAACCATTTCTTATCTTTTTTTTAAGAAAAAATATGGTAGACTAACTGATCTTTCGATCAGTTAATGAAAGAGCCCAATGCAAAAAAAAATGCATGTTGGGTCTTTGAAAGAGTTCGAATCATTTTGATAATAATAAGTTCGAACTCTTTTACCGAGCAGGTCTACGGTTCGAGTCCGTATAGCCCTA

atpF (ATCG00130)

TTAATCAGTTATTTCTTTCATCGTACCAAACATCCCAATATTTGCATTAATAGTACGTAAATGTAACTCATTACTCAAACAACTATTTAGGGTTCCTATAGCTCCTTGTAAAGCTTGTTGGAAAACCCGTTCGCGGACTTGATTAATTGTTCTTTGTTGCTCAAAAAGAATGGTTTCATTTTTGTAATTTTCTAATTGTTTCAAAGTTTTATAAGTTGAATTAATCAAATTCAATTTTTCTCGTTCGATTTCAGAGTATCCATTCACGCGAAACTTATCCGCTTCCGTTTCTACGTTACGCAAGCGCGCCCGGGCATTTTCTAATTGTTGAATAGCTCCTTCACGCAGTTCTTCTGAATTTCGAATAGTATTTAATATCCTCTGCTTTCGGTTATCTAATAAATCATTTAATGAAAGTAGATTATCTTTCCATTCATTAAAAAAAAAGTTCTATGATCCCTTCCCGAACCAAACATGAATCTTTCGATTCATTTGGCTCTCATGCTCACTTATTCCAATCAATTATTTATGAGACTTCCCTTCCCATCTTTTTCATGCAATGAGCCTACCCTCTCCCGAGTTTTTTTATTCAATTCATATTCAATATATATTTCTATCGAAAAAGATCACCAATCCAAGATAAAAATATTCGGAGGATTCTTCTGACCAATGAAAAATTAATAATTGTCAGCAAAGTTGTTTCTTTTTTTCTTGAAATCCAAAGAATTCTTATTATTTTATACGTAGGTTATCAATTCTGCATTATAAAAAAAGACTCAAAAAATTTTATCGACATGAGTGTTTTATATCGAAAAAAGTATAACAATTCTTTTTGAAAATTTTATTCATTTTTTTATTTAGTCTACATTTAGACTATATATAGTAGAAAGAGTACCATGTTGCATCTGAACTTCAAACGGTTTAGCTTTAACCATGTTAATTAATTGTCCCAATTTTTGGTTAATAGAGAATCAAAGTAAAGCGGACTTACCAAAGAATAACGAAAGGCTATGGTTCTCAAATATGATTTTTTTATTCAGAAGTAATTCGCGGGATTATGCACTCTTTCCTAGTTATAGTGCCACTGGGTGAATCCAGCCTATTCTTGAAATGAACAACTCACACACACTCCCTTTCCAAAAAAGATCAATACACCGAAAACTACACTTAGATTTATTGGATTTGTTGCTAAAATATCGGTATTAAATCCGAAACTCCCGGCGGATGGCCAGTGACCCAAGTAAACGAAAGAATCGGTTAAATTTTTCAT

rps12 (ATCG00065 ATCG00905)

ATACACCCGAGTACATGTTCCTCGTCGCTGAGGGCATCCCCGAAGCGCTGGGGATTTCGTGACGTTTCGGATTGGCTGTCTTGTATTTCTAATAAGTTGTTTAATGGTTGGCATTTATXXXXXXXX…TTTGGCTTTTTGACCCCATATTGTAGGGTGGATCTCGAAAGATATGAAAGATCTCCCTCCAAACCGTACATACGACTTTCATCGAATACGGCTTTCCACAGAATTATATATGTATCTCTGAAATCGAGTATGGAATTCTGTTTACTCACTTTTAAATTGAGTATCCGTTTCCCTCCTTTTCCTGCTAGGATTGGAAATCCTGTATTTTACATATCCATACGATTGAGTCCTTGGGTTTCCGAAATAGTGTAAAAATAAGTGCTTCGAATCATTGCTATTTGACCCGGACCTGTTCTAAAAAAGTCGAGGCATTTCGAATTGTTTGTTGACACGGACAAAGTCAGGGAAAACCTCTGAAATTATTTCAATATTGAACCTTGGACATATAAGAGTTCCGAATCGAATCTCTTTTGAAAGAAGATCTTTTGTCTCGTGGTAGCCTGCTCCAGTCCCCTTACGAAACTTTCGTTATTGGGTTAGCCATACACTTCACATGTTTCTAGCGATTCACATGGCATCATCAAATGATACAAGTCTTGGATAAGAATCTACAACGCACTAGAACGCCCTTGTTGACGATCCTTTACTCCGACAGCATCTAGGGTTCCTCGAACAATGTGATATCTCACACCGGGTAAATCCTTAACCCTTCCCCCTCTTACTAAGACTACAGAATGTTCTTGTAAATTATGGCCAATACCAGGTATATAAGCAGTGATTTCAAATCCCGAGGTTAATCGTACTCTGGCAACTTTACGTAAAGCAGAGTTTGGTTTTTTGGGGGTGATAGT

Rps12-2-int1

Rps12-2-int2

rpl2 (ATCG00830)

CTATTTACTACGGCGACGAAGAATCAAAGTCTCACTATATTTTTTCCTTTTTCTAGTTCTTCTTCCAAGTGCAGGATAACCCCAGGGGGTTACGGGTTTTTTTCTACCAATTGGAGCCCTCCCTTCACCACCTCCATGGGGATGGTCGACAGGGTTCATAACTACTCCTCTTACTACAGGACGTTTACCTAGCCAACATTTCGATCCGGCTCTACCCAAACTTTTCTGGTTTACCCCAACATTTCCCACTTGTCCGACTGTTGCTGAGCAGTTTTTGGATATCAAACGGACCTCTCCAGAAGGTAATTTTAATGTGGCCGATTTCCCCTCTTTTGCAATCAGTTTCGCTACAGCACCCGCTGCTCTAGCTAATTGTCCACCCCTTCCAAGTGTGATTTCTATATTATGTATGGCCGTGCCTAAGGGCATATCGGTTGAAGTAGATTCTTCTTTTTGATCAATCAAAACCCCTTCCCAAACTGTACAAGCTTCTTCCAAAGCATACGGCTTTCTGGATGTAGATGATGATATCTATACGGATGGATCTTATATATATCGTAGAATTCTTCTATATATGGTAGAAGTACCACACGAGTGGATATATAGGAATCAAAATCTGCCGAATAACTTATGTTATGATCTTCTACATCCTAGGTCTTCCCGTTCCGTCATCTGGCTTATGTTCTTCATGTAGCATTCAGACCGAATGACTCTATGAAATTACGTCGATACTTCCACATATTATGGGTAACGTAGGAGACATCTCTATTTTTCCCCGGGGGAATCTTTAGAATTACCACTGCTTAGCTTTCAATTCGCCTCTGACCATCAAATGAAATGTGAATAACCCGTCCTCCTCTCTTTGAAACAAGGGGCGCTTATGGTTCTGTCGGTGCTTGAAACAATTTTGTCTTCTCCATATTACTATATCTCTAGAGTCAATAATTTTATATGAGGAACTACTGAACTCAATCACTTGCTGCCGTTACTCTTCAGTTTTCTGTTGAGGTCTATCCTGTAGAGGTACTCAAATTGGATCAGTGATCGATTTCTAGGTTTCGTCGTAAACCTAATTGGTTACTTCCAATTACGTAAATCAAATAGTTCAAACCGCACTCAAAGGTAGGGCATTTCCCATTTTTATAGGAACTTCTGTACCAGAAACAATGGTATCTCCAATTATAGCCCCTCTGGGATGTAAAATATATCTCTTCTCACCATCCCCATAGTGTATGAGACAAATGTATGCATTTCGATTAGGGTCGTATTCTATGGTTACGATTCTACCATATATGTCTTTTGCATTTCGTCGAAAATCTATTTTACGGTATAGACGCTTATGACCTCCCCCTCTATGCCTTGCGGTAATTATTCCTCTGGCATTACGACCTTTACCACAATGATGCTGCCCACAGATCAAATTATTTCGTGGATTGGATTTCACTTGACTGTCTACGGCTCCATTGCGTGTGCTCGGGGTAGAAGTTTTGTATAAATGTATCGCCAT

trnA (ATCG00940)

GGGGATATAGCTCAGTTGGTAGAGCTCCGCTCTTGCAATTGGGTCGTTGCGATTACGGGTTGGGTGTCTAATTGTCCAGGCGGTAATGATAGTATCTTGTACCTGAACCGGTGGCTCACTTTTTCTAAGTAATGGGGAAAAGGACCGAAACATGCCACTGAAAGACTCTACTGAGACAAAGATGGGCTGTCAAGAACGTAGAGGAGGTAGGATGGTCAGTTGGTCAGATCTAGTATGGATCGTACATGGACGGTAGTTGGAGTCGGCGGCTCTCCTAGGGTTCCCTCGTCTGGGATTGATCCCTGGGGAAGAGGATCAAGTTGGCCCTTGCGAACAGCTTGATGCACTATCTCCCTTCAACCCTTTGAGCGAAATGCGGCAAAAGGAAGGAAAATCCATGGACCGACCCCATCGTCTCCACCCCGTAGGAACTACGAGATCACCCCAAGGACGCCTTCGGTATCCAGGGGTCGCGGACCGACCATAGAACCCTGTTCAATAAGTGGAATGCATTAGCTGTCCGCTCGCAGGTTGGGCAGTAAGGGTCGGAGAAGGGCAATCACTCATTCTTAAAACCAGCATTCGAAAGAGTTGGGGCGGAAAAGGGGGGGAAAGCTCTCCGTTCCTGGTTCTCCTGTAGCTGGATCCTCTAGAACCACAAGAATCCTTAGTTGGAATGGGATTCCAGCTCATCACCTTTTGAGATTTTGAGAAGAGTTGCTCTTTGGAGAGCACAGTACGATGAAAGTTGTAAGCTGTGTTCGGGGGGGAGTTCTTGTCTATCGTTGGCCTCTATGGTAGAATCAGTCAGGGGCCTGATAGGCGGTGGTTTACCCTGTGGCGGATGTCAGCGGTTCGAGTCCGCTTATCTCCA
