# Supplementary figures and images for: A PPR Protein ACM1 Is Involved in Chloroplast Gene Expression and Early Plastid Development in Arabidopsis
Source: Int J Mol Sci. 2021 Mar 3;22(5):2512. doi: 10.3390/ijms22052512 (PMC7959153; doi:10.3390/ijms22052512)

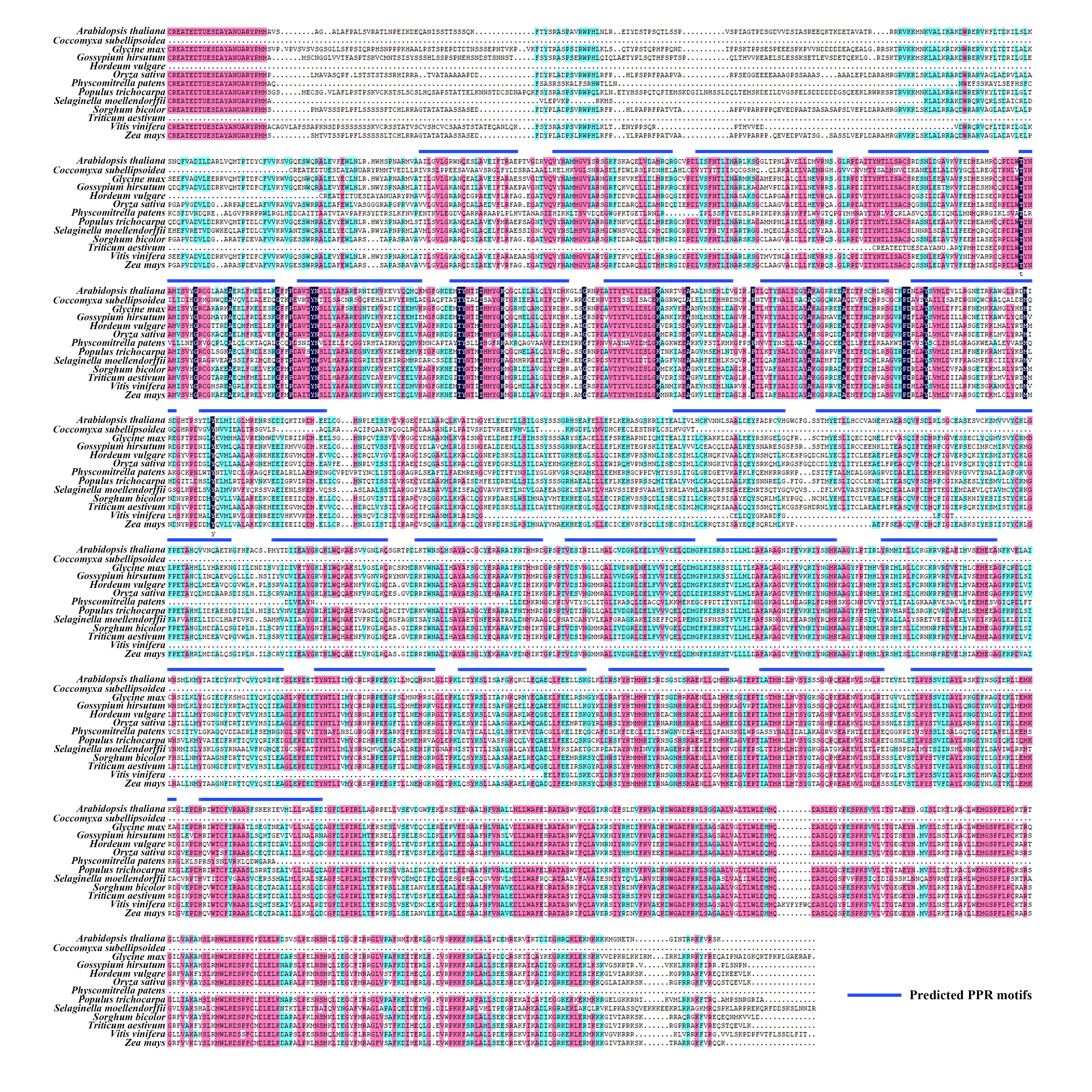

Supplement: Supplementary file 1 [file ijms-22-02512-s001.zip › Figure S1.jpg]
